# Residual Change of Four Pesticides in the Processing of *Pogostemon cablin* and Associated Factors

**DOI:** 10.3390/molecules28186675

**Published:** 2023-09-18

**Authors:** Yuanxi Liu, Zuntao Zheng, Hongbin Liu, Dongjun Hou, Hailiang Li, Yaolei Li, Wenguang Jing, Hongyu Jin, Ying Wang, Shuangcheng Ma

**Affiliations:** 1 Institute for Control of Chinese Traditional Medicine and Ethnic Medicine (ICCTMEM), National Institutes for Food and Drug Control (NIFDC), Beijing 100050, China; liuyuanxi@nifdc.org.cn (Y.L.);; 2 Institute for the Control of Agrochemicals, Ministry of Agriculture and Rural Affairs, Beijing 100125, China; 3 China Animal Disease Control Center, Ministry of Agriculture and Rural Affairs, Beijing 102629, China

**Keywords:** residual change, processing factor, *Pogostemon cablin*, volatile oil, UPLC-MS/MS, traditional Chinese medicine

## Abstract

Before use as medicines, most traditional Chinese medicine (TCM) plants are processed and decocted. During processing, there may be some changes in pesticide residues in TCM. In recent years, reports have studied the changes of pesticides during the processes of boiling, drying and peeling of TCM materials but have rarely involved special processing methods for TCM, such as ethanol extraction and volatile oil extraction. The changes of carbendazim, carbofuran, pyridaben and tebuconazole residues in common processing methods for *P. cablin* products were systemically assessed in this study. After each processing step, the pesticides were quantitated by UPLC-MS/MS. The results showed amount decreases in various pesticides to different extents after each processing procedure. Processing factor (PF) values for the four pesticides after decoction, 75% ethanol extraction and volatile oil extraction were 0.02~0.75, 0.40~0.98 and 0~0.02, respectively, which indicated that residual pesticide concentrations may depend on the processing technique. A risk assessment according to the hazard quotient with PF values showed that residual pesticide amounts in *P. cablin* were substantially lower than levels potentially posing a health risk. Overall, these findings provide insights into the safety assessment of *P. cablin*.

## 1. Introduction

*Pogostemon cablin* (Blanco) Benth. (*P. cablin*) belongs to the family *Lamiaceae*, which is native to southeast Asian areas, and has seen extensive cultivation in Indonesia, Malaysia, China and Brazil [1]. As a very important traditional Chinese medicine, *P. cablin* (“Guanghuoxiang” in Chinese) is utilized for removing dampness, regulating gastrointestinal function and relieving superficies syndrome [2]. *P. cablin* is known to comprise multiple constituents such as monoterpenoids, triterpenoids, sesquiterpenoids, phytosterols and flavonoids [3]. Currently, studies analyzing *P. cablin* for composition have focused on the volatile oil, also termed patchouli volatile oil, revealing multiple constituents, including patchouli alcohol (PA), pogostone, α-patchoulene, β-patchoulene, and β-elemene [4]. PA, the most abundant constituent [5], possesses multiple bioactivities such as anti-peptic ulcer, anti-microbial, anti-oxidative and anti-inflammatory activities, as well as immune and intestinal microbiome regulatory effects. Products containing *P. cablin* are widely used; specifically, patchouli volatile oil is used not only in medicine but also in fragrance manufacturing [6] and as a food supplement [7]. Patchouli volatile oil has been commonly used as a base for cosmetic formulations. Additionally, it is widely applied, thanks to multiple bioactivities such as anti-oxidant, anti-depressant, anti-inflammatory, analgesic, cytotoxic and anti-microbial effects [8]. According to the *Chinese Pharmacopoeia*, there are about 50 main preparations that contain *P. cablin* currently sold, including pills, tablets, granules, capsules and oral liquids.

Assessing pesticide residues in *P. cablin* is very important to ensure the medicinal safety of *P. cablin* and to establish appropriate regulatory strategies. However, studies evaluating pesticide residues in *P. cablin* are rare, focusing on detecting organochlorine or organophosphorus, rather than involving the assessment of multi-pesticidal residues in *P. cablin*. Thus, it is of great importance to establish guidelines for the safe use of pesticides through the study of pesticide residues and health risk assessment. In recent years, the processing factor (PF) has attracted increasing attention in the risk assessment of pesticides. At the same time, studies have shown that using PF values may have a great impact on risk assessment results. In general, PF is defined as the effect and disposition on pesticide residues during processing [9]. It is calculated as the residual levels in processed products by the amounts in raw materials. PF may be more meaningful and could provide a more accurate basis for risk assessment when the residue concentrations change during the processing steps. It is known that routine processing steps, including washing, peeling, blanching, juicing, fermentation and distillation, could decrease the food amounts of pesticide residues [10,11]. Hakme et al. [12] studied the PF values during baking for 41 pesticides in cereal bran-based biscuits. As a result, PF values for most pesticides were in the range of 1 to 0.76. However, for polar constituents, including carbendazim and volatile components, a PF of 0.67 was obtained. Bai et al. [13] studied 18 pesticides in field-collected rice specimens during Chinese Baijiu production. It was found that the residues of the 18 pesticides in the rice were significantly reduced by the process of Chinese Baijiu production and their PF values were generally lower than 1. The processing of traditional Chinese medicine (TCM) is different from that of food. At present, research on how pesticides in TCM change during processing has mainly focused on drying and decocting [14,15]; there are only a few studies regarding other processing methods. For TCM with high volatile oil content, such as *P. cablin* and perilla, alcohol extraction products or volatile oil are used as medicine. However, volatile oil extraction, one of the important processing methods for TCM, has not been examined for its effects on pesticide amounts.

As the test TCM in this study, we chose *P. cablin,* which has been widely used in many formulations. Pesticides were selected based on the literature research, suggestions from farmers and monitoring results of pesticides in TCM plants, which can be representative of the pesticides used in the growth of *P. cablin*.

The purpose of this study was to establish an LC-MS/MS technique for determining multiple pesticides in *P. cablin* and to evaluate the pesticide intake risk, considering processing factors. The processing factors involved in volatile oil extraction from Chinese medicinal materials were studied for the first time. The results could provide a reference for accurately evaluating the safety of *P. cablin* and formulating pesticide monitoring strategies.

## 2. Results and Discussion

### 2.1. Method Validation

In recent years, determination methods for pesticides in *P. cablin* have been reported. For example, Nie et al. [16] established a modified QuEChER technique combined with gas chromatography/mass spectrometry (GC-MS/MS) for the detection of 107 pesticides; d-SPE tubes with 900 mg anhydrous MgSO_4_, 900 mg PSA, 300 mg C_18_, 300 mg silica and 90 mg GCB were used to clean up the samples. Meanwhile, Yang et al. [17] optimized a GC-FPD protocol for simultaneously analyzing 44 organophosphorus pesticides in *P. cablin*; the QuEChER protocol was also utilized in this study for sample preparation, with d-SPE tubes containing 60 mg PSA sorbent, 20 mg GCB and 120 mg anhydrous MgSO_4_. However, research on the pesticide residues of *P. cablin* mostly focused on nonpolar to lowly polar pesticides detected by GC or GC-MS/MS, while polar pesticides detected by LC-MS/MS have been rarely examined.

Here, a LC-MS/MS protocol for pesticide detection in *P. cablin* was established. *P. cablin* contains a large amount of volatile substances and pigments, which may interfere with the detection of pesticide residues. Selecting appropriate pretreatment and detection methods to avoid the interference of the matrix effect should be carried out as much as possible to ensure the accuracy of results. At present, the SPE method can effectively separate pesticides and organic contaminants in a complex matrix. Zhou et al. [18] developed a reliable protocol for quantitating 43 pesticides in *Schizonepeta tenuifolia* cleaned with Pesticarb/NH_2_ cartridges. Li et al. [19] screened for 439 pesticides in fruits and vegetables with Carb/NH_2_ cartridges and obtained satisfactory results. In the present work, we purified samples with Carb/NH_2_ cartridges, which have a strong purification ability. This method can remove most of the pigments from *P. cablin* extract.

The linearity of our LC-MS/MS assay was assessed with six calibration points, utilizing matrix-matched standards with a concentration range of 0.5 to 100 μg·kg^−1^, which can reduce the matrix effect. Because there is little difference in the matrix effect for the four pesticides in the processed samples, *P. cablin* was used for method validation. Pesticide recovery levels in *P. cablin* were between 75.3% ± 4.97 and 103.5% ± 6.33 after spiking with 10, 50 and 250 μg·kg^−1^. The instrument limits of detection were 5~10 μg·kg^−1^ for all four compounds. Six-point calibration standards were injected at 10~100 μg·kg^−1^ for verifying instrument calibration; a regression coefficient (r^2^) of >0.990 for the calibration curve was obtained. Validation parameters for the determination of the four pesticides are shown in Table 1.

### 2.2. Pesticides Detected in Samples

Recently, *P. cablin* extracts combined with various herbs have been used widely to improve rheumatism, release the exterior, remove phlegm and regulate stomach functions. However, pesticide residues in *P. cablin* have been rarely studied and the few existing reports mainly focus on the detection of organophosphorus and organochlorine pesticides. For example, Yang et al. [17] studied 44 organophosphorus pesticide residues in 44 *P. cablin* batches and associated products (e.g., *P. cablin*, *P. cablin* oil and powder) by GC-MS/MS. As a result, chlorpyrifos was detected in one PO and one *P. cablin* at 0.024 and 0.036 mg·kg^−1^, respectively, which were reduced compared with MRL amounts (0.05 mg·kg^−1^). Wu et al. [20] quantitated 9 organochlorine pesticides (DDTs, BHCs and pentachloronitrobenzene) in 60 *P. cablin* batches collected from 3 planting bases. The results showed that the residual levels of BHC in *P. cablin* ranged from 4.95 to 25.58 μg.kg^−1^; total residual amounts of DDT were 8.93~12.48 μg.kg^−1^.

In the present study, we originally selected 137 pesticides as detection objects. With increasing attention paid to herbal formulae, safety and toxicity concerns have been raised. A total of 33 banned pesticides, most of which are highly toxic and/or carcinogenic, have been documented in the latest version of the *Chinese Pharmacopoeia*. In this study, in addition to the banned pesticides that can be assessed by LC-MS/MS, some commonly used pesticide indicators, including carbendazim, chlorpyrifos, acetamiprid and so on, were also considered as detection indicators. A total of 137 pesticide residues in 9 batches of *P. cablin* from different sources were detected by LC-MS/MS. The sample detection results are shown in Table 2.

There were four pesticides detected in nine batches of *P. cablin*, including carbendazim, pyridaben, pyraclostrobin and carbofuran. The results showed high detection rates for carbendazim, pyridaben and pyraclostrobin (more than 30%), which was confirmed by El-Sheikh et al. [21] when determining the pesticide residues in vegetable and fruits. These three pesticides are widely used in medicinal plant cultivation due to high efficiency and low toxicity. Although the detection rate of carbofuran was low, it is a highly toxic pesticide and should also be paid attention to. These four pesticides have been used in *P*. *cablin* planting and are easily left over.

### 2.3. Effects of Processing on Residual Amounts of Pesticides

Research indicates that routine processing steps, including washing, peeling, blanching, juicing, fermentation and distillation, decrease the food amounts of pesticide residues [22]. For instance, Yang and colleagues analyzed various pesticides and two degradation products in soil, seed, peanut, oil and dregs’ specimens. They found that, compared with peanuts, the total masses of the majority of pesticides in oil and shell specimens were reduced, but both degradation products, including λ-cyhalothrin and pirimicarb (3-phenoxybenzoic and desmethyl-formamido- pirimicarb), were higher, which indicated the degradation products continued to be generated during pressing. M.A. Cámara [23] found that, during the industrial processing of canned apricot and peach, washing/cutting and canning had the highest impacts in lowering pesticide residues with PF values of less than 0.6. For orange juice production, squeezing reduced the residues the most, with PF > 0.6.

However, processing methods differ between TCM and food. From raw medicinal materials to products administered to humans, most TCMs need to go through treatment steps such as water decoction, ethanol extraction and so on. Reports have studied the changes of pesticides in the process of TCM processing. Xiao et al. [14] first reported the effects of various processing techniques on PF values for Radix Paeoniae Alba. The results showed that TCM processing could partially remove a variety of pesticide residues, with a removal rate of 98%. Noh et al. [24] assessed residual buprofezin amounts in freshly collected ginseng and determined their level changes during processing. The results showed that residual amounts rose with ginseng processing into dried and red ginseng. He et al. [25] assessed the behaviors of tebuconazole, prochloraz and abamectin residues in Rehmannia during decoction processing. The results showed that the pesticide residues were markedly reduced upon washing, carbonizing and boiling, with PFs below 1 during processing. Xiao et al. [15] investigated the impacts of drying (oven drying, sun drying and shade drying) and decocting on 11 pesticides in honeysuckle and reached the conclusion that, although drying substantially increased pesticides’ concentration effect and induced the generation of multiple pesticide metabolites, the corresponding decoction remarkably decreased the risk of pesticide residues to human health. It is noteworthy that existing reports mainly focused on the impacts of drying and decoction on residual pesticide levels in TCM. However, for *P. cablin,* which contains large amounts of volatile oil, the most widely used method is to extract the volatile oil from its raw material and prepare it into a patented medicine or use it in health products and cosmetics.

According to the *Chinese Pharmacopoeia*, there are about 50 main preparations that contain *P*. *cablin* currently sold, including pills, tablets, granules, capsules, oral suspensions and so on. The preparation process for *P. cablin* is summarized, mainly including extracting the volatile oil into the medicine, water decoction and 75% ethanol extraction. Among them, adding the extracted patchouli volatile oil into the preparation is the most widely used processing method. However, the changes in pesticide residues during the extraction of patchouli volatile oil have not been reported. Finally, the changes in detected pesticides under three common processing methods for *P. cablin* have been studied. The PF values of the three processing methods are shown in Table 3.

**Figure 1:**
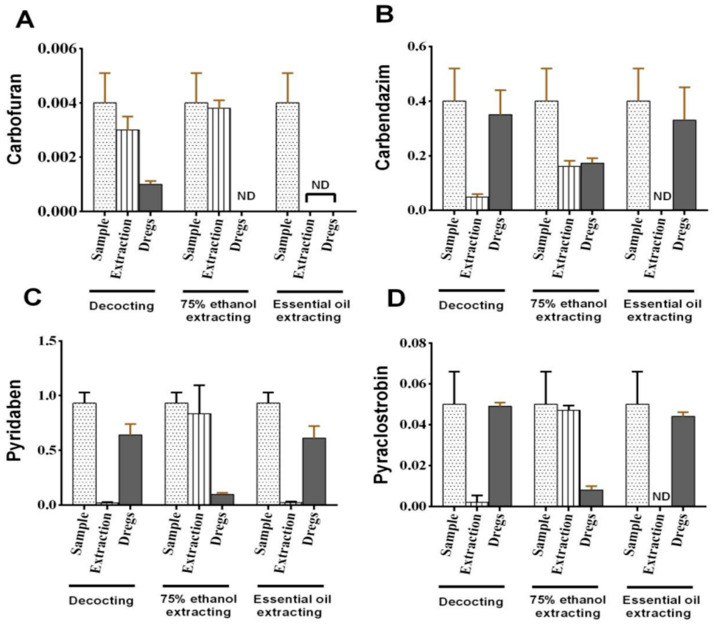
Pesticide residues in *P. cablin* under different processing methods. (**A**) Carbendazim; (**B**) pyridaben; (**C**) pyraclostrobin; (**D**) carbofuran.

The results showed that, after the whole processing procedure, the PF values of the four pesticides in the decoction, 75% ethanol extract and volatile oil extraction were 0.02~0.75, 0.40~0.98 and 0~0.02, respectively. The PF values were greater for the four pesticides obtained by 75% ethanol extraction compared with the other two methods. Especially for carbofuran, the PF value with 75% ethanol extraction reached 0.98, indicating almost all carbofuran in *P. cablin* was transferred into the 75% ethanol extraction. However, PF values for both boiling and volatile oil processing were far less than 1, which indicates that the four pesticides were hardly transferred to the end products in these two processing methods (Figure 1). Moreover, changes in these four pesticides during boiling were very different. For example, the PF value for carbofuran in the process of boiling reached 0.75, while PF values for the remaining three pesticides were less than 0.12. The PF values obtained for pesticides were different, which was closely related to their physicochemical properties, particularly water solubility and water octanol partition coefficient [26].

### 2.4. Processing Factors and Dietary Exposure Assessment

In a previous study, we used the HQ method for evaluating the risk of pesticide residues in 10 TCM preparations [27]. The HQ of each pesticide was calculated as a ratio of exposure amounts to an acceptable level, e.g., ADI or acute reference dose (ARfD). Moreover, the years and frequency of taking TCM have been considered in the risk assessment of pesticide residues in TCM, making the risk assessment results closer to the real situation. Recently, this method has been broadly used to assess exposure to pesticide residues in TCM. However, few studies have considered the impact of PF value on risk assessment results. In this study, the effects of PF were considered in the exposure assessment of pesticides, especially for specific processing methods of *P. cablin* such as volatile oil extraction.

Here, risk assessment according to the hazard quotient with PFs showed that exposure to pesticide residues in *P. cablin* was substantially reduced compared with allowable levels in humans. However, when PF values were not considered, the risk caused by *P. cablin* pesticides was obviously overestimated. As shown in Table 3, the HQ values of pyridaben and pyraclostrobin in *P. cablin* without decocting treatment were 50 and 25 times higher than those obtained after decoction treatment, respectively. However, the decoction process converted pesticide residues into compounds with higher toxicity, such as 3,5,6-trichloropyridinol (the main chlorpyrifos metabolite); therefore, its metabolites should be evaluated in further risk assessment.

## 3. Materials and Methods

### 3.1. Standards and Chemicals

Pesticide standards (carbendazim, carbofuran, pyraclostrobin and pyridaben) were provided by the Ministry of Agriculture (Beijing, China) and Dr. Ehrenstorfer GmbH (Augsburg, Germany), with >96% purity. They were solubilized in acetone at 100 µg·mL^−1^ and kept at −20 °C until use. Dilutions of pooled standard solutions were made in acetonitrile.

The Carb/NH_2_ SPE cartridge (500 mg, 6 mL) was provided by Agela Technologies (Shanghai, China). Analytical-grade sodium chloride, glacial acetic acid and solvents were from Sinopharm Chemical Reagent (Beijing, China). HPLC-grade acetonitrile and acetone were provided by Fisher Scientific (Waltham, MA, USA).

### 3.2. Sample Preparation and Processing

**Figure 2:**
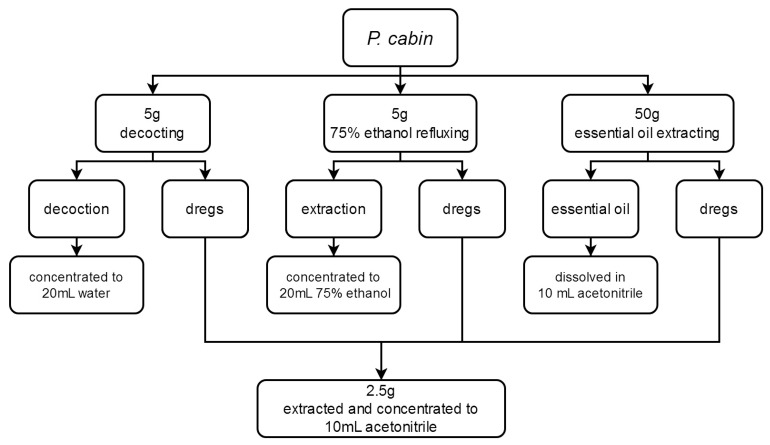
*P. cablin* processing flow diagram.

A total of 9 different *P. cablin* specimens were obtained in herbal markets and identified by Associate Professor Wenguang Jing at the National Institute for Food and Drug Control, China. Sample 6, in which 4 representative pesticides were detected, was used to investigate processing factors. According to its use in TCM decoction and proprietary Chinese medicine, *P. cablin* was processed in 3 different ways, as shown in Figure 2.

In this study, both the extraction solvent and the dregs were obtained for investigating residual changes for pesticides during *P. cablin* processing and kept in a hermetically closed container at −20 °C. The pesticides were detected within one day. The detailed production processes were:

Process 1. Decocting. A total of 5 g of the sample was decocted with 100 mL water (100 °C, boiling) for 1 h. The decoction was concentrated at 45 °C to near-dryness and redissolved in water (20 mL).

Process 2. Seventy-five percent ethanol extraction. A total of 5 g of the sample was extracted with 100 mL 75% ethanol at 85 °C for 1 h under reflux. The extract was concentrated to near-dryness at 45 °C and redissolved in 75% ethanol (20 mL).

Process 3. Essential oil extraction. A total of 50 g of the sample was extracted by steam distillation (100 °C, boiling) for 5 h and the essential oil was dissolved in 10 mL acetonitrile.

Dregs’ processing: the dregs of each processing method were collected and dried separately at 60 °C for 24 h for the next step of pesticide extraction.

### 3.3. Extraction and Purification

Method for extraction: An amount of 4.0 mL extract in acetonitrile:toluene 3:1 (*v*/*v*) was loaded into a Carb/NH_2_ column at 1 mL·min^−1^. Elution was performed with 20 mL of acetonitrile:toluene 3:1 (*v*/*v*). The obtained eluate underwent evaporation at 40 °C to about 3~5 mL. The final residue was redissolved in 10.0 mL acetonitrile for further assessment.

Method for herbal residues: 2.5 g of the processed herbal residue (powder) was mixed with 1.0 g sodium chloride and 50 mL acetonitrile and the mixture underwent homogenization (2 min) and centrifugation (4000 rpm for 5 min). The resulting supernatant was collected and 50 mL acetonitrile was added to the remaining residue for a second extraction (homogenization and centrifugation). Both supernatants were pooled and evaporated at 40 °C to about 1~2 mL. The resulting residue was redissolved in 10.0 mL acetonitrile; the purification steps were consistent with those of the above extraction.

### 3.4. LC-MS/MS Analytical Conditions

Specimens were analyzed on a Waters Acquity UPLC equipment coupled with a XEVO Triple Quad mass spectrometry system (Waters Co., Milford, MA, USA). Specimens injected at 1 μL were separated on an ACQUITY UPLC BEH C18 column (2.1 × 100 mm; Waters), with an eluent consisting of solution A (5 mM ammonium formate in water, containing 0.1% formic acid) and solution B (5 mM ammonium formate in 95% methanol, containing 0.1% formic acid). A flow rate of 0.4 mL·min^−1^ was utilized with the following gradient: 0~0.8 min, 30% B; 0.8~11.0 min, 30~100% B; 11.0~13.0 min, 100% B; 13.0~14.0 min, 100~30% B; 14.0~18.0 min, 30% B. A drying gas flow rate of 8 L·min^−1^ and an oven temperature of 30 °C were applied. The multiple reaction monitoring (MRM) mode was utilized for detection in the electrospray positive ionization mode (ESI+). Source temperature, declustering potential, desolvation gas flow rate, cone gas flow rate and desolvation temperature were 150 °C, 30 V, 900 L/Hr, 50 L/Hr and 500 °C, respectively. In our previous study, 141 pesticides in *P. cablin* were screened. Finally, it was found that the detection rates of 3 pesticides were higher than 30%, including carbendazim, pyraclostrobin and pyridaben. One banned pesticide (carbofuran) was also found in *P. cablin.* Therefore, the PF values of these four pesticides during the processing of *P. cablin* were studied. The MRM features of these 4 pesticides are listed in Table 4 and those of another 137 pesticides are shown in Table S1, based on previous methods described by our team [28].

### 3.5. Determination of Processing Factors and Hazard Quotients

The PF, reflecting the effectiveness of pesticide removal, is determined as residue levels in the processed product by those of the raw (unprocessed) material, which is obtained based on Equation (1).

(1) $$PF=\frac{\mathrm{Residual}\;\mathrm{levels}\;\mathrm{of}\;\mathrm{processed}\;\mathrm{products}}{\mathrm{Residual}\;\mathrm{levels}\;\mathrm{of}\;\mathrm{raw}\;\mathrm{materials}}$$

*PF* < 1 indicates reduced pesticide residue amounts post-processing; d *PF* > 1 points to a concentration effect of the processing technique [29].

The hazard quotient (HQ) value was utilized for assessing the risk of dietary exposure. HQ is the ratio of exposure level to health guidance value (acceptable daily intake (ADI) or acute reference dose (ARfD)). *HQ* < 1 and *HQ* > 1 reflect acceptable and unacceptable risk levels, respectively, the higher the value, the higher the risk. Considering substantial differences between TCM and food products, exploring the key factors of risk assessment of pesticides in TCM via multiple questionnaires associated with consumption features of TCM, the consumption frequency and exposure time of TCM were obtained and applied to the evaluation of chronic exposure [27], calculated from Equation (2):

(2) $$HQ=\frac{R\times CR\times EF\times Ed\times PF}{bw\times AT\times ADI}$$

where *R* represents the mean residue levels of the pesticide in the specimen (mg·kg^−1^); *CR* represents the mean commodity consumption (kg·day^−1^) (ChP 2020); *EF* represents exposure frequency, i.e., 90 days/year according to previously published methods; *Ed* is exposure time, i.e., 20 years based on the questionnaire results [30,31]; *AT* represents average time, i.e., life expectancy (365 day/year × 70 years); *bw* represents the mean adult body weight in Chinese individuals (63 kg). The obtained dietary exposure estimate was compared to relevant toxicological references (e.g., *ADI*) for the given pesticide.

### 3.6. Statistical Analysis

Triplicate measurements are mean ± standard deviation.

## 4. Conclusions

This study clearly demonstrated changes in four pesticide residues in commonly applied methods for *P. cablin* processing. The removal of pesticide residues was affected by processing steps to different extents. After the whole processing procedure, the concentrations of these pesticides were lower, with PF < 1 after every processing method. The PF values for the four pesticides in the decoction, 75% ethanol extract and volatile oil were 0.02~0.75, 0.40~0.98 and 0~0.02, respectively. The PF values for both boiling and volatile oil were far less than 1, which indicated that the four pesticides were hardly transferred to the end products using these two processing methods. Moreover, the changes in the four pesticides during these processes differed, which may be closely related to the physicochemical features of the pesticides, especially their water solubility and water octanol partition coefficient. Overall, risk assessment according to the hazard quotient with PFs demonstrated exposure to pesticide residues in *P. cablin* was substantially below the amounts potentially posing a health risk. Processing factors are very important for accurate pesticide risk assessment in TCM and further related in-depth and extensive research is warranted. In addition, this study paid attention to the transfer of pesticides during volatile oil extraction from *P. cablin*, which provided a reference for the safety evaluation of volatile oil products in TCM. However, certain limitations were also noted. The *P. cablin* sample was processed in the laboratory and the pesticide residues in sample were low, which may have led to a gap between the results we obtained and the results of actual production in a factory. In subsequent research, the targeted spraying of these pesticides during the cultivation of *P. cablin* will be carried out, as well as large-scale factory production research, to prepare for the formulation of maximum residue limits for pesticides.

## Figures and Tables

**Table 1:** Validation parameters for the determination of 4 pesticides.

Processing	Pesticide	r	LOD (mg·kg^−1^)	Recovery/%		Precision, RSD/%
				Mean	RSD	
*P. cablin*	Carbendazim	0.9908	<0.005	101.06	5.84	3.16
	Carbofuran	0.9996	<0.005	103.50	6.33	2.11
	pyraclostrobin	0.9997	<0.01	83.57	5.68	6.09
	pyridaben	0.9936	<0.005	75.30	4.97	1.58

**Table 2:** Pesticide residues detected in 9 batches of *P. cablin* (mg·kg^−1^).

Pesticide	S1	S2	S3	S4	S5	S6	S7	S8	S9
Carbendazim	ND	ND	ND	0.01	0.04	0.40	0.01	ND	0.09
Carbofuran	ND	ND	ND	ND	ND	0.01	0.02	0.004	ND
Pyridaben	ND	ND	ND	0.01	ND	0.93	0.01	0.01	ND
Pyraclostrobin	0.02	ND	ND	ND	0.27	0.05	ND	ND	ND

ND: not detected.

**Table 3:** Processing factors and hazard quotients for four *P. cablin* pesticides.

Pesticides	*PFs*		HQ	HQ × *PF*	Overestimated Value
Carbendazim	Decocting	0.12	5.66 × 10^−5^	0.68 × 10^−5^	8.33
	75% ethanol extracting	0.40		2.26 × 10^−5^	2.50
	Essential oil	/		/	
Carbofuran	Decocting	0.75	1.37 × 10^−4^	1.03 × 10^−4^	1.33
	75% ethanol extracting	0.98		1.34 × 10^−4^	1.02
	Essential oil	/		/	
Pyridaben	Decocting	0.02	2.96 × 10^−4^	0.06 × 10^−4^	50
	75% ethanol extracting	0.90		2.66 × 10^−4^	1.11
	Essential oil	0.02		0.06 × 10^−4^	50
Pyraclostrobin	Decocting	0.04	3.38 × 10^−5^	0.14 × 10^−5^	25
	75% ethanol extracting	0.94		3.18 × 10^−5^	1.06
	Essential oil	/		/	

**Table 4:** MRM parameters of the four pesticides.

Pesticide	Retention Time t (min)	Cone Voltage (V)	Quantitative Ion	CE1	Qualitative Ion	CE2
Carbendazim	1.23	30	192.1 > 160.1	18	192.1 > 132.1	28
Carbofuran	3.49	20	222.1 > 123.0	21	222.1 > 165.1	16
Pyraclostrobin	7.33	30	388.1 > 194.1	17	388.1 > 296.1	19
Pyridaben	9.83	2	365.2 > 147.1	24	365.2 > 309.2	12

## Data Availability

The datasets generated during and/or analyzed during the current study are available from the corresponding author on reasonable request.

## Supplementary Materials

The following supporting information can be downloaded at: https://www.mdpi.com/article/10.3390/molecules28186675/s1, Table S1: MRM parameters of the 126 pesticides.
