# Supplementary material for: Residual Change of Four Pesticides in the Processing of Pogostemon cablin and Associated Factors
Source: Molecules. 2023 Sep 18;28(18):6675. doi: 10.3390/molecules28186675 (PMC10535192; doi:10.3390/molecules28186675)
Supplement: Supplementary file 1 [file molecules-28-06675-s001.zip › molecules-2585627-supplementary.pdf]

Supplementary Materials

Residual changes in four pesticides in the processing of Pogo-stemon cablin and associated factors

Table.S1 MRM parameters of the 126 pesticides

No.	Pesticide	Retention time t/min	Mode	Cone voltage / V	Quantitative ion	CE1	Quanlitative ion	CE2
1	nitenpyram	1.17	ESI+	22	271. 1＞125. 9	25	271. 1＞224. 9	12
2	monocrotophos	1.17	ESI+	20	224. 1＞127. 0	22	224. 1＞193. 0	11
3	pymetrozine	0.94	ESI+	44	218. 0＞105. 0	16	218. 0＞78. 9	28
4	omethoate	0.96	ESI+	30	214. 0＞183. 0	15	214. 0＞155. 0	21
5	Aldicarb sulfoxide	0.97	ESI+	30	207. 1＞89. 0	14	207. 1＞132. 0	10
6	Dinotefuran	1.07	ESI+	12	203. 0＞129. 0	12	203. 0＞157. 0	8
7	propamocarb hydrochloride	0.95	ESI+	30	189. 1＞102. 0	17	189. 1＞144. 0	12
8	cyromazine	0.91	ESI+	66	167. 0＞60. 1	18	167. 0＞85. 3	18
9	Daminozide	0.92	ESI+	2	161. 1＞143. 1	10	161. 1＞102. 2	16
10	Methamidophos	0.96	ESI+	30	142. 0＞93. 9	13	142. 0＞124. 9	13
11	thiamethoxam	1.41	ESI+	28	292. 1＞211. 0	10	292. 1＞132. 0	22
12	Trichlorfon	1.53	ESI+	30	256. 9＞109. 0	18	256. 9＞79. 0	30
13	Aldicarb sulfone	1.24	ESI+	30	223. 1＞86. 0	14	223. 1＞148. 0	10
14	thiabendazole	1.33	ESI+	30	202. 0＞175. 0	35	202. 0＞131. 0	45
15	Chlordimeform	1.39	ESI+	10	197. 1＞46. 2	18	197. 1＞117. 1	24
16	Hymexazol	1.2	ESI+	34	100. 1＞54. 2	14	100. 1＞44. 2	12
17	Imidacloprid	1.8	ESI+	30	256. 1＞	20	256. 1＞	15

					175. 1		209. 1	
18	Phosfolan	2.12	ESI+	17	256. 0＞ 140. 0	20	256. 0＞ 168. 0	20
19	Clothianidin	1.66	ESI+	15	250. 0＞ 169. 0	17	250. 0＞ 132. 0	12
20	Carbofuran-3-hy droxy	1.7	ESI+	30	238. 1＞ 181. 1	10	238. 1＞ 163. 0	16
21	Flonicamid	1.54	ESI+	32	230. 0＞ 203. 0	16	230. 0＞ 148. 0	24
22	Acetamiprid	1.94	ESI+	30	223. 0＞ 126. 0	20	223. 0＞ 56. 1	15
23	Diethyl aminoethyl hexanoate	1.82	ESI+	24	216. 1＞ 143. 1	16	216. 1＞ 100. 0	14
24	tricyclazole	1.98	ESI+	30	190. 0＞ 163. 0	28	190. 0＞ 136. 0	34
25	nicosulfuron	2.42	ESI+	30	411. 0＞ 182. 0	22	411. 0＞ 182. 0	32
26	flumetsulam	2.14	ESI+	37	326. 1＞ 129. 0	25	326. 1＞ 109. 0	50
27	phosphamidon	2.5	ESI+	30	300. 1＞ 174. 1	14	300. 1＞ 127. 1	25
28	imazethapyr	2.32	ESI+	40	290. 0＞ 230. 0	18	290. 0＞ 247. 8	23
29	sulfoxaflor	2.49	ESI+	20	308. 2＞ 174. 2	12	308. 2＞ 154. 1	29
30	thiacloprid	2.4	ESI+	32	253. 0＞ 126. 0	20	253. 0＞ 90. 1	40
31	Difenzoquat-met hyl-sulfate	2.17	ESI+	50	249. 2＞ 130. 1	55	249. 2＞ 193. 2	40
32	ethiofencarb	1.7	ESI+	30	239. 1＞ 72. 0	18	239. 1＞ 182. 1	15
33	thidiazuron	2.64	ESI+	28	221. 0＞ 101. 9	15	221. 0＞ 93. 9	13
34	aldicarb	2.62	ESI+	19	213. 1＞ 89. 1	16	213. 1＞ 116. 1	11
35	cymoxanil	2.29	ESI+	30	199. 0＞ 128. 0	8	199. 0＞ 111. 0	18
36	Cinosulfuron	3.16	ESI+	27	414. 0＞ 183. 0	24	414. 0＞ 157. 0	16
37	Thifensulfuron-m ethyl	2.94	ESI+	25	388. 0＞ 167. 0	15	388. 0＞ 56. 0	40
38	Metsulfuron methyl	3.12	ESI+	30	382. 0＞ 167. 0	16	382. 0＞ 198. 9	22

39	Thiodicarb	3.18	ESI+	20	355. 0＞87. 9	20	355. 0＞107. 9	16
40	Thiophanate-methyl	3.1	ESI+	30	343. 0＞151. 0	22	343. 0＞93. 0	46
41	mesotrione	2.93	ESI+	23	340. 1＞228. 1	18	340. 1＞104. 0	32
42	imazalil	3.21	ESI+	31	297. 0＞259. 0	22	297. 0＞69. 0	22
43	bentazone	3.08	ESI+	30	241. 1＞199. 1	12	241. 1＞107. 2	26
44	Simetryn	3.16	ESI+	35	214. 0＞124. 0	20	214. 0＞95. 9	25
45	Indolebutyric acid	3.07	ESI+	60	204. 2＞186. 0	17	204. 2＞144. 0	30
46	simazine	2.94	ESI+	32	202. 0＞124. 0	17	202. 0＞96. 0	22
47	metolcarb	2.99	ESI+	30	166. 0＞109. 0	12	166. 0＞94. 1	27
48	Rimsulfuron	3.44	ESI+	27	431. 9＞182. 1	22	431. 9＞325. 1	14
49	Ethametsulfuron-methyl	3.62	ESI+	30	411. 2＞168. 1	30	411. 2＞196. 1	15
50	flumorph	3.91	ESI+	30	372. 3＞285. 2	20	372. 3＞165. 1	30
51	Florasulam	3.37	ESI+	29	360. 0＞129. 0	50	360. 0＞108. 9	22
52	chlorsulfuron	3.43	ESI+	30	358. 0＞141. 1	16	358. 0＞167. 0	18
53	ehaboxam	3.81	ESI+	30	321. 1＞183. 0	20	321. 1＞200. 0	22
54	flutriafol	3.68	ESI+	23	302. 1＞70. 2	18	302. 1＞123. 1	29
55	fosthiazate	3.77	ESI+	30	284. 1＞104. 0	32	284. 1＞228. 0	14
56	phorate sulfoxide	3.66	ESI+	15	277. 0＞96. 9	32	277. 0＞143. 0	20
57	Propoxur	3.4	ESI+	12	210. 0＞111. 0	16	210. 0＞168. 0	10
58	Isoproturon	3.94	ESI+	18	207. 1＞72. 1	14	207. 1＞165. 1	12
59	Carbaryl	3.72	ESI+	30	202. 1＞145. 1	22	202. 1＞117. 1	28
60	Chlorantraniliprole	4.68	ESI+	20	481. 9＞283. 9	14	481. 9＞450. 8	20

61	bensulfuron methyl	4.41	ESI+	30	411. 1＞149. 0	22	411. 1＞182. 0	20
62	ethiprole	4.98	ESI+	27	397. 1＞351. 1	25	397. 1＞255. 1	9
63	Tribenuron	4.5	ESI+	30	396. 1＞154. 9	14	396. 1＞180. 9	22
64	dimethomorph	4.84	ESI+	10	388. 0＞301. 0	18	388. 0＞165. 1	30
65	Paclobutrazol	4.86	ESI+	30	294. 1＞70. 2	20	294. 1＞125. 1	38
66	phorate sulfone	4.88	ESI+	30	293. 0＞171. 0	16	293. 0＞247. 0	9
67	demeton	4.44	ESI+	30	259. 0＞89. 0	10	259. 0＞61. 0	33
68	clomazone	4.71	ESI+	23	240. 0＞125. 0	18	240. 0＞89. 0	46
69	diuron	3.98	ESI+	30	233. 0＞72. 1	30	233. 0＞46. 3	14
70	isoprocarb	4.26	ESI+	30	194. 1＞95. 1	14	194. 1＞137. 1	8
71	pyrazosulfuron-ethyl	5.13	ESI+	22	415. 0＞82. 9	45	415. 0＞139. 0	48
72	Chlorimuron-ethyl	5.34	ESI+	42	414. 9＞185. 8	18	414. 9＞83. 0	50
73	Spirotetramat	5.16	ESI+	30	374. 3＞216. 2	20	374. 3＞302. 4	30
74	Epoxiconazole	5.54	ESI+	30	330. 0＞121. 0	22	330. 0＞101. 0	50
75	phosmet	5.33	ESI+	30	318. 0＞160. 0	22	318. 0＞77. 0	46
76	methidathion	5.12	ESI+	30	303. 0＞145. 0	10	303. 0＞85. 1	20
77	Uniconazole	5.47	ESI+	45	292. 0＞69. 8	35	292. 0＞124. 9	35
78	Myclobutanil	5.41	ESI+	30	289. 1＞125. 1	32	289. 1＞70. 2	18
79	diethofencarb	5.23	ESI+	30	268. 0＞226. 0	10	268. 0＞124. 0	40
80	prometryn	5.27	ESI+	27	242. 0＞158. 0	24	242. 0＞200. 1	17
81	Fenobucarb	5.04	ESI+	30	208. 0＞94. 9	14	208. 0＞152. 0	8
82	Spinosad	5.62	ESI+	30	732. 4＞142. 2	25	732. 4＞98. 1	15

83	mandipropamid	5.59	ESI+	16	412. 3＞328. 2	16	412. 3＞356. 2	10
84	Methoxyfenozide	5.96	ESI+	20	369. 1＞149. 1	20	369. 1＞313. 2	10
85	Flusilazole	5.92	ESI+	30	316. 1＞247. 1	18	316. 1＞165. 1	28
86	diflubenzuron	5.97	ESI+	2	311. 1＞158. 1	14	311. 1＞113. 1	48
87	tebuconazole	5.86	ESI+	14	308. 2＞70. 1	18	308. 2＞125. 1	36
88	Fenhexamid	5.66	ESI+	32	302. 1＞97. 2	22	302. 1＞55. 3	38
89	Bifenazate	5.93	ESI+	7	301. 1＞198. 0	20	301. 1＞107. 0	10
90	mefenacet	5.59	ESI+	16	299. 0＞120. 0	25	299. 0＞148. 0	15
91	Tridemorph	6.02	ESI+	50	298. 1＞130. 0	25	298. 1＞98. 0	32
92	Napropamide	5.88	ESI+	21	272. 1＞129. 1	16	272. 1＞171. 1	18
93	triflumuron	6.86	ESI+	30	359. 0＞156. 1	16	359. 0＞139. 1	35
94	clodinafop-propargyl	7.05	ESI+	25	350. 0＞91. 0	32	350. 0＞266. 0	16
95	triflumizole	7.18	ESI+	2	346. 3＞278. 0	8	346. 3＞69. 1	6
96	Zoxamide	7.17	ESI+	27	336. 0＞187. 1	25	336. 0＞159. 0	40
97	quinalphos	6.86	ESI+	30	299. 0＞147. 1	20	299. 0＞163. 1	20
98	cadusafos	7.28	ESI+	30	271. 1＞159. 0	16	271. 1＞131. 0	22
99	emamectin benzoate	6.74	ESI+	30	886. 8＞82. 2	60	886. 8＞158. 2	21
100	Carfentrazone-ethyl	6.74	ESI+	56	412. 2＞346. 0	22	412. 2＞366. 0	16
101	Rotenone	6.3	ESI+	46	395. 0＞192. 1	24	395. 0＞213. 1	24
102	prochloraz	6.3	ESI+	6	375. 9＞308. 0	14	375. 9＞266. 0	14
103	Flufenacet	6.34	ESI+	16	364. 0＞152. 1	23	364. 0＞194. 1	11
104	Tebufenozide	6.59	ESI+	30	353. 1＞133. 0	20	353. 1＞297. 1	8

105	Cyazofamid	6.72	ESI+	17	325. 0＞107. 9	20	325. 0＞261. 0	10
106	hexaconazole	6.12	ESI+	12	314. 1＞159. 1	30	314. 1＞70. 1	18
107	isazofos	6.65	ESI+	30	314. 0＞162. 1	16	314. 0＞120. 0	28
108	Edifenphos	6.57	ESI+	23	311. 0＞109. 0	32	311. 0＞111. 0	26
109	iprobenfos	6.11	ESI+	9	289. 0＞91. 0	20	289. 0＞205. 0	10
110	Penconazole	6.16	ESI+	46	284. 0＞70. 1	20	284. 0＞159. 1	28
111	famoxadone	7.16	ESI+	12	392. 2＞331. 1	10	392. 2＞238. 0	20
112	coumaphos	7.32	ESI+	30	363. 0 > 227. 0	35	363. 0 > 307. 0	22
113	pencycuron	7.5	ESI+	48	329. 0＞125. 0	22	329. 0＞218. 0	14
114	sulfotep	7.5	ESI+	30	323. 0＞171. 0	15	323. 0＞97. 0	32
115	Diazinon	7.4	ESI+	30	305. 1＞169. 1	20	305. 1＞97. 0	32
116	clofentezine	7.43	ESI+	19	303. 0＞138. 0	18	303. 0＞102. 0	33
117	Fonofos	7.49	ESI+	30	247. 1＞109. 0	20	247. 1＞137. 0	10
118	indoxacarb	7.78	ESI+	30	528. 1＞528. 1	22	528. 1＞203. 0	40
119	benfuracarb	8.43	ESI+	14	411. 1＞195. 0	23	411. 1＞190. 0	13
120	trifloxystrobin	7.91	ESI+	32	409. 0＞186. 1	16	409. 0＞145. 0	44
121	Diflufenican	7.77	ESI+	14	395. 0＞266. 1	22	395. 0＞246. 2	34
122	haloxyfop(free acid)	7.7	ESI+	30	376. 0＞316. 0	25	376. 0＞288. 0	35
123	Oxaziclomefone	8.51	ESI+	40	376. 0＞190. 0	12	376. 0＞161. 0	26
124	Phosalone	7.6	ESI+	30	367. 9＞181. 9	14	367. 9＞110. 9	42
125	Clethodim	8.36	ESI+	30	360. 1＞164. 1	23	360. 1＞268. 1	14
126	Chlorfluazuron	9.07	ESI+	33	539. 8＞382. 9	20	539. 8＞158. 0	20

127	bromadiolone	8.87	ESI+	2	527. 3＞152. 2	56	527. 3＞251. 3	26
128	(E)-Fenpyroximate	9.14	ESI+	30	422. 2＞366. 1	15	422. 2＞138. 1	32
129	hexythiazox	8.93	ESI+	30	353. 0＞228. 1	14	353. 0＞168. 1	26
130	chlorpyrifos	8.91	ESI+	2	350. 0＞97. 1	26	350. 0＞198. 0	18
131	Pyriproxyfen	8.86	ESI+	20	322. 0＞96. 1	14	322. 0＞185. 0	24
132	buprofezin	8.87	ESI+	30	306. 2＞201. 1	15	306. 2＞116. 1	20
133	Diafenthiuron	9.46	ESI+	30	385. 3＞329. 3	17	385. 3＞236. 2	49
134	propargite	9.28	ESI+	20	368. 2＞231. 2	10	368. 2＞175. 1	14
135	Etoxazole	9.25	ESI+	30	360. 3＞141. 1	20	360. 3＞113. 1	30
136	Triallate	9.39	ESI+	23	304. 0＞142. 9	18	304. 0＞86. 0	28
137	amitraz	9.84	ESI+	52	294. 1＞163. 1	16	294. 1＞122. 1	30
